# Research framework for food security and sustainability

**DOI:** 10.1038/s41538-025-00379-x

**Published:** 2025-01-31

**Authors:** Effie Papargyropoulou, John Ingram, Guy M. Poppy, Tom Quested, Clara Valente, Lee Ann Jackson, Tim Hogg, Thom Achterbosch, Emanuele Paolo Sicuro, Susanne Bryngelsson, Isabelle Guelinckx, Peter Van Dael, Louise Dye

**Affiliations:** 1 Sustainability Research Institute, School of Earth and Environment, University of Leeds, Leeds, UK; 2 Environmental Change Institute, University of Oxford, Oxford, UK; 3 University of Southampton, Southampton, UK; 4 WRAP, Second Floor, Blenheim Court, 19 George Street, Banbury, Oxon OX16 5BH UK; 5 NORSUS, Norwegian Institute for Sustainability Research, Stadion 4, 1671 Kråkerøy, Norway; 6 OECD, Paris, France; 7 Universidade Católica Portuguesa, Porto, Portugal; 8 Wageningen University, Wageningen, The Netherlands; 9 Cargill, Vilvoorde, Belgium; 10 RISE, Research Institutes of Sweden, Gothenburg, Sweden; 11 ILSI Europe, Brussels, Belgium; 12 InnoNext Ltd., Montreux, Switzerland; 13 School of Food Science and Nutrition, University of Leeds, Leeds, UK

**Keywords:** Interdisciplinary studies, Sustainability

## Abstract

This article presents a framework for food security and sustainability research, developed by industry, academia, and public sector experts. Key priorities for collaborative research include reassessing food system contexts and drivers, adapting food system activities, transforming food system outcomes, developing and applying food system methodologies, and adopting an ethical and just lens. The framework emphasises the need for coordinated action across multiple scales and sectors, focusing on synergies and trade-offs as opposed to isolated food activities, to address complex challenges in food security and sustainability.

## Introduction

Ensuring that all people at all times have physical, economic and social access to sufficient, safe, nutritious food to meet their dietary needs and food preferences for an active and healthy life and doing so while respecting planetary boundaries is one of the biggest challenges facing humanity^1–3^. To this end, ILSI Europe convened a workshop on the 17^th^ November 2022 comprising 15 industry, academic, and public sector experts on the topic of ‘Food Security and Sustainability’. The aim of the workshop was to *‘identify, define and prioritise themes within the field of Food Security and Sustainability to which coordinated public-private collaborative research can make a significant contribution’*. This *perspective* article reports on the workshop’s findings and critically discusses the implications for the scientific community and the food sector.

The article is organised as follows: the Approach section covers the methodology, data collection, and analysis techniques used. The next two sections present the proposed framework for food security and sustainability research developed, and summarise the priority research questions identified. The article concludes by discussing the framework’s contributions, applications, limitations, and implications for the scientific and broader food community.

## Approach

A combined Nominal Group Technique (NGT) and Focus Group approach was taken to achieve consensus amongst the participants of the multi-stakeholder workshop in *identifying, defining and prioritising themes within the field of Food Security and Sustainability to which coordinated public-private collaborative research can make a significant contribution*. NGT uses structured small group discussion to generate and prioritise ideas, and to achieve group consensus often on contentious topics^4^. It has been widely applied in health, social services, education, and strategic planning^5^. Incorporating a focus group within the NGT, allowed to combine the prioritisation process of a standard NGT with the in-depth discussion of the focus group. The process involved 6 phases:

Phase 1 - Introduction and Explanation: The facilitator explained the purpose of the session and the process to the participants.

Phase 2 - Silent Idea Generation: Participants were asked to write down their ideas independently and silently in response to the prompt of: *Identify themes and research questions within the field of Food Security and Sustainability to which coordinated public-private collaborative research can make a significant contribution*. These were recorded and shared on the real-time online collaborative platform Padlet.

Phase 3 - Round-Robin Sharing: Each participant shared one idea (research theme or question) at a time in a round-robin format, which was recorded by the facilitator. This continued until all ideas are shared.

Phase 4 – Focus Group Discussion: The group discussed each idea to clarify and define them. This step ensured that everyone understood the topics and research questions presented and could ask questions or provide feedback. Phases 3 and 4 happened concurrently.

Phase 5- Voting and Ranking: Participants privately voted on the most pressing topics and research questions presented. Participants could vote on one or more of the research topics and questions recorded. The facilitator then tallied the votes to identify the most voted topics and questions.

Phase 6 - Final Prioritisation: The results of the exercise were discussed, and a final list of prioritised ideas were created based on the group’s input.

Following the workshop, the research topics identified, defined and prioritised were subsequently thematically analysed and grouped under five categories adapted from Ingram and Thornton’s framework on *Transforming Food Systems Outcomes*^6^. A deductive thematic analysis of the qualitative data collected during the workshop (i.e. the transcribed group discussions, and research topics and questions generated) provided a focused and efficient way of data analysis^7^ and it involved the following phases:

Phase 1 - Predefined Framework: Ingram and Thornton’s framework on *Transforming Food Systems Outcomes* was established as the most suitable framework to guide the qualitative data analysis, due to its comprehensive nature and focus on transformation of food systems outcomes.

Phase 2 - Data Familiarisation: the researchers immersed themselves in the data to understand the context and content.

Phase 3 - Applying Codes: Using the predefined themes, the researchers systematically coded the data i.e. looked for instances in the data that fit the established themes.

Phase 4 - Reviewing and Refining Themes: The coded data was reviewed to ensure it aligned with the predefined themes. Although the thematic analysis was primarily deductive in nature (i.e. built around the existing themes from Ingram and Thornton’s framework), to avoid excluding research topics and questions that did not fit within the predefined framework, two new themes were added to the existing framework. The workshop participants were given the opportunity to shape the outputs from Phase 4, through an iterative feedback process.

Phase 5 - Interpreting Data: The final step involved interpreting the data within the context of the predefined and newly developed themes, providing insights that are grounded in the conceptual framework.

An ethical review was completed before commencing data collection, in accordance with the University of Leeds Research Ethics and Integrity Framework. In line with this framework, the identity of the research participants was kept anonymous and participant consent protocols were followed.

## Framework for food security and sustainability research

**Fig. 1:**
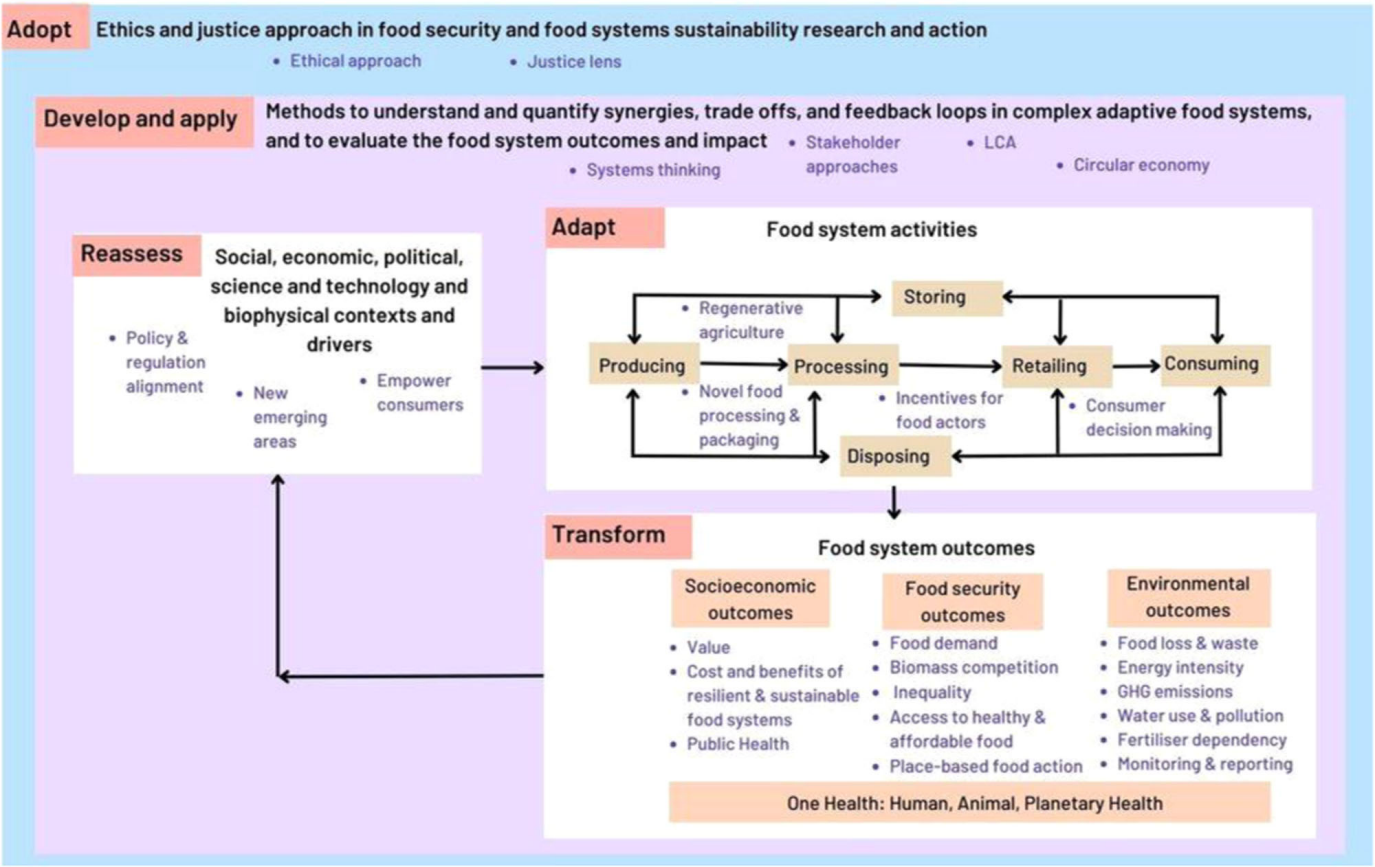
Framework for food security and sustainability research. Adapted from Ingram and Thornton^6^. The framework consists of five themes (i to v below), each containing research topics of the highest priority (in blue font bullet points), which in turn include key research questions summarised in Table 1.

The resulting framework for food security and sustainability research is summarised in Fig. 1 below. The framework consists of five themes (*i* to *v* below), each containing research topics of the highest priority (in blue font bullet points), which in turn include key research questions. The themes, topics and research questions are discussed in the sections below, and the key research questions are also summarised in Table 1 for ease of use.

**Table 1:** Summary of priority questions on food security and sustainability research, under the five thematic categories of the research framework presented in Fig. 1

**i) (Re)assess food system contexts & drivers**
1. How can novel technologies help achieve food security and sustainability goals? 2. What are the impacts of novel technologies on food security, nutrition and public acceptability? 3. How can food, farming, environment, health, economic policies be better aligned for a more integrated approach to food systems decision making?
**ii) Adapt food system activities**
4. How can best practice in regenerative agriculture be captured and shared across different contexts to i) promote evidence-based practice and ii) enable scaling up and/or scaling out? 5. What type of incentives can empower food actors to prioritise, adopt and maintain regenerative agriculture practices? 6. How can food processing support food security and sustainability outcomes? 7. How can food processing improve nutritional quality of foods by improving the bioavailability of nutrients? 8. How can consumers make informed decisions that support sustainability, nutritional and food security?
**iii) Transform food system outcomes**
**Food Security outcomes**
9. How can we reduce food demand? 10. How can we rebalance biomass demand for food and energy production? 11. What role more localised food systems play in food security and sustainability? 12. How can placed-based food systems (e.g. community supported agriculture, social supermarkets, food surplus redistribution, living labs) be scaled out to deliver food security and environmental, societal and economic co-benefits? 13. How can we improve access and availability of healthy and affordable food in cities to address household level food insecurity and inequality? 14. How can sustainable food consumption also be affordable?
**Environmental outcomes**
15. How can we better measure food waste at the farm? 16. How can food losses and waste monitoring and reporting be improved globally? 17. What behaviour change models, interventions and strategies can be effective in reducing household food waste? 18. How can we better align food losses and waste interventions at different supply chain stages to maximise synergies? 19. What role can valorisation play in food losses and waste reduction? 20. How can phosphorus use become more efficient and sustainable across the food, agriculture, waste sectors to improve food security and sustainability outcomes? 21. How can we reduce GHG emissions, soil degradation, biodiversity loss, water use and pollution linked to food system activities?
**Socio-economic outcomes**
22. How can we quantify the externalised social costs (e.g. costs of ill health due to poor diets to public health services) and environmental costs (e.g. cost of pollution, climate change, deforestation, biodiversity loss) of food production and consumption to make economic case for action? 23. Are sustainable and/or resilient food systems less profitable? Over what timescale should we investigate this?
**iv) Develop and apply food system approaches and methodologies**
24. What are the implications for nutrition of the circular economy approach? 25. How can we incorporate more social and economic criteria in Life Cycle Assessment? 26. How can we evaluate and increase supply chain resilience to physical, social and political shocks? 27. How can we better balance local and global supply chains to increase food supply chains resilience?
**v) Adopt ethics and a justice lens**
28. In food security and sustainability research and action: What is right? What is fair? And for whom?

### (Re)assess food system contexts & drivers

The first theme involved the social, economic, political, science and technology, and biophysical contexts within which food systems operate and the drivers that influence food actors’ behaviour. Important research topics grouped under this theme involved the role of technology, governance and policy (both as contexts and drivers for change) in influencing and responding to food consumers and industry, and ultimately shaping possible pathways towards food security and sustainability.

New and emerging areas such as alternative protein sources, novel food processing technologies e.g. 3-D printing, and precision agriculture were identified as topics of interest: *how can novel technologies help achieve food security and sustainability goals, and what are their impacts in terms of food security, nutrition and public acceptability?* The need for evidence-based policy and better alignment across relevant policies, regulations, governance levels and geographies, consistent with the One Health approach, was considered a topic of urgency: *how can food, farming, environment, health, economic policies be better aligned for a more integrated approach to food systems decision making*? The role of industry and regulatory bodies in empowering the consumer to make healthier and more sustainable choices (beyond voluntary standards and information campaigns), was highlighted as a priority topic for research. Central to all these discussions was the question of possible pathways to change, and the role of food actors in coordinated, synergistic action at multiple scales to bring about change addressing complex ‘wicked’ challenges.

### Adapt food system activities

This theme included food producing, processing and packaging, distributing and retailing, and consuming activities by relevant food system actors. Discussions grouped under this theme focused on regenerative agriculture and novel processing as means to achieve food security and sustainability. It was recognised that regenerative agriculture has been advancing in recent years, and better knowledge sharing is essential to bring regenerative agriculture practice into the mainstream. *How can best practice in regenerative agriculture be captured and shared across different contexts to i) promote evidence- based practice and ii) enable scaling up and/or scaling out? What type of incentives can empower food actors to prioritise, adopt and maintain regenerative agriculture practices?* The role of processing and packaging in food security and sustainability was also considered important, for example extending shelf life can reduce food waste and greenhouse gases and improve access to safe and, in some cases, nutritionally dense food^8^. However, the possible negative impacts of food processing, for example reduced nutritional content of some processed foods, also need to be monitored, as well as consumers’ perceptions affecting acceptability. *How can food processing support food security and sustainability outcomes? How can food processing improve nutritional quality of foods by improving the bioavailability of nutrients? How can consumers make informed decisions that support sustainability, nutritional and food security?*

### Transform food system outcomes

This theme involved food security outcomes i.e. food access (e.g. affordability, allocation and preference), food availability (e.g. production, distribution, exchange) and food utilisation (e.g. nutritional value, social/cultural value, food safety). Food system outcomes also include broader socioeconomic (e.g. livelihoods, wealth, social, political and human capitals) and environmental outcomes (e.g. Green House Gas (GHG) emissions, impacts on biodiversity and water quality). Environmental and planetary health was considered as cutting across the socio-economic, food security and environmental outcomes. The workshop’s discussions focused on how food system outcomes can be transformed to achieve food security and sustainability (via steps i. and ii. above), the most appropriate scale for action, and how the impact could be evaluated.

Topics under food security outcomes included the need to manage – and not just only strive to meet – demand, the role more localised food systems can play in terms of self-sufficiency and sustainability, the role of cities and urban food systems as ‘agents of change’, and the importance of equality and food justice in achieving both household level food security and sustainability. It was agreed that research and action towards food security has focused on increasing food production, whereas managing (i.e. reducing) demand has lacked attention. *How can we reduce food demand? How can we rebalance biomass demand for food and energy production?* Participants also highlighted how recent environmental, geopolitical and socio-economic shocks to the food system, demonstrated the need to reconsider the important role that more localised food systems can play in food security (particularly in terms of self-sufficiency), and sustainability (e.g. in reducing GHG emissions of the globalised food system offering all year-round fresh produce supply). *What role do more localised food systems play in food security and sustainability*? Likewise, there was a renewed focus on the role urban food systems can play in reducing household level food insecurity and carbon emissions, while simultaneously delivering other co-benefits (e.g. community cohesion, food resilience, healthy diets, inclusive growth). *How can placed-based food systems (e.g. community supported agriculture, social supermarkets, food surplus redistribution, living labs) be scaled out to deliver food security and environmental, societal and economic co-benefits?* Finally, it was emphasised that food security and sustainability outcomes cannot be achieved without prioritising equality and food justice, recognising that access and affordability are key components of food security and sustainability. *How can we improve access and availability of healthy and affordable food in cities to address household level food insecurity and inequality? How can sustainable food consumption also be affordable?*

Topics affecting environmental outcomes included food loss and waste (FLW), energy intensity and fertiliser dependency of the food system. Preventing FLW at all stages of the food supply chain was considered essential both for food security and sustainability^9^. Food waste at the farm and household are key priority areas in terms of improved reporting, and prevention interventions beyond knowledge campaigns^10^. *How can we better measure food waste at the farm? How can FLW monitoring and reporting be improved globally? What behaviour change models/interventions can be effective in reducing household food waste? How can we better align FLW interventions at different supply chain stages to maximise synergies? What role can valorisation play in FLW reduction*? The current cost of living and energy crisis was considered an opportune moment to reduce energy and FLW along the food supply chain. On the other hand, food systems depend on fertilisers, causing aquatic pollution and making the food system vulnerable to supply disruptions and price fluctuations^11^. One example is phosphorus-based fertilisers. There is no substitute for phosphorus in food production and all food systems are now dependent on fertilisers derived, in large part, from a finite supply of phosphate rock. *How can phosphorus use become more efficient and sustainable across the food, agriculture, waste sectors to improve food security and sustainability outcomes?*

Discussions on socio-economic outcomes predominantly focused on the costs and benefits of resilient and sustainable food systems, and how these could act as effective drivers for change. Participants reflected on the ‘value’ we place on desirable food systems outcomes such as human and planetary health, the costs we assign to undesirable outcomes such as food insecurity and environmental destruction, and how these relate to the profitability of food. *How can we quantify the externalised social costs (e.g. costs of ill health due to poor diets for public health services) and environmental costs (e.g. cost of pollution, climate change, deforestation, biodiversity loss) of food production and consumption to make an economic case for action? Are sustainable and/or resilient food systems less profitable, and over what timescale should we investigate this?* It was also considered important to reevaluate what society deems as acceptable food system outcomes in the context of affordability and cost of living crisis.

### Develop and apply food system approaches and methodologies

This theme included research approaches, methodologies and tools to i) analyse and ii) evaluate food systems in order to iii) inform strategies and interventions. The topics grouped under this theme included the use of stakeholder engagement and circular economy approaches, Life Cycle Assessment (LCA) methodologies and tools in food supply chains^12^.

Stakeholder engagement approaches were considered important to understand and balance values, interests and power across food actors. It was also considered important for LCA methodologies and circular economy approaches to broaden their environmental focus and capture more of the social and economic aspects of sustainability^13^: *what are the implications for nutrition of the circular economy approach? how can we incorporate more social and economic criteria in LCA?* Resilience was deemed vital to achieving food security, as were tools to assess strengths and weaknesses in local and global food supply chains: *How can we evaluate and increase supply chain resilience to physical, social and political shocks? How can we better balance local and global supply chains to increase food supply chains resilience?*

### Adopt an ethical and a just lens

The final theme reflected the need for food actors and those involved in shaping and delivering research in food security and sustainability, to be aware and committed to issues of ethics and justice^14^. When engaging in research and action aiming to address these two connected challenges, it is imperative to be attentive to questions concerning ‘*What is right? What is fair? And for whom?’*. Food actors and researchers are embedded within the food system, and as a result they are active and influential participants with agency and power.

## Priority research questions on food security and sustainability

The previous section discussed the themes, topics and research questions organised under the proposed framework for food security and sustainability research. For ease of use, Table 1 below summarises the priority research questions identified in this study. These can be used to guide collaborative research between food actors and form the basis of funding proposals and research designs addressing food security and sustainable challenges.

## Proposed framework contribution, applications, and limitations

The proposed framework has the potential to steer future research efforts in food security and sustainability. It supports transdisciplinary research by identifying specific research themes and questions where collaboration between public and private food systems actors can make a meaningful contribution. It also directs food stakeholders on key research priorities that can form the foundation of funding proposals and research designs. Policymakers can use the framework to align food, farming, environment, health, and economic policies. This alignment can help create a more integrated approach to food systems decision-making.

Although the food experts participating in this exercise brought a primarily European perspective, food security and sustainability is a global challenge and most of the themes and questions generated are relevant in other geographical and socio- economic contexts. Furthermore, the methodological approach adopted in identifying the key research priorities can be applied in other contexts as well, to generate more localised outputs if needed.

The framework for food security and sustainability research integrates expertise from both the private and public sectors within the food system. However, further work is required in validating the framework through case studies and pilot applications, to further refine and enhance its rigor. Demonstrating the framework’s value and effectiveness in these ways would strengthen the argument for its adoption.

## Conclusion

The research topics in food security and sustainability discussed above, have implications for the scientific and broader food community. The ultimate aim of this research is to transform the food system outcomes in order to achieve food security and sustainability (see section iii.) For this transformation to happen it is necessary to (re)assess how contexts and drivers affect ‘signals’ that drive food systems behaviour (section i.) and to adapt food system activities of food actors (section ii). To this end, researchers can develop and apply methods to understand and quantify synergies, trade-offs and feedback loops within the complex adaptive food systems and evaluate the food system outcomes and impact (section iv), while adopting an ethics and food justice lens in food security and food systems sustainability research and action (see section v). To achieve meaningful progress in food security and sustainability, these two interconnected challenges need to be addressed simultaneously, with collaboration and coordinated action across all food actors. In this context ILSI Europe can drive the proposed research agenda forward by convening collective action by the relevant food actors.
